# Increased Hippocampal Excitability in the 3xTgAD Mouse Model for Alzheimer's Disease *In Vivo*

**DOI:** 10.1371/journal.pone.0091203

**Published:** 2014-03-12

**Authors:** Katherine E. Davis, Sarah Fox, John Gigg

**Affiliations:** Faculty of Life Sciences, University of Manchester, Manchester, United Kingdom; University of Lancaster, United Kingdom

## Abstract

Mouse Alzheimer's disease (AD) models develop age- and region-specific pathology throughout the hippocampal formation. One recently established pathological correlate is an increase in hippocampal excitability in vivo. Hippocampal pathology also produces episodic memory decline in human AD and we have shown a similar episodic deficit in 3xTg AD model mice aged 3–6 months. Here, we tested whether hippocampal synaptic dysfunction accompanies this cognitive deficit by probing dorsal CA1 and DG synaptic responses in anaesthetized, 4–6 month-old 3xTgAD mice. As our previous reports highlighted a decline in episodic performance in aged control mice, we included aged cohorts for comparison. CA1 and DG responses to low-frequency perforant path stimulation were comparable between 3xTgAD and controls at both age ranges. As expected, DG recordings in controls showed paired-pulse depression; however, paired-pulse facilitation was observed in DG and CA1 of young and old 3xTgAD mice. During stimulus trains both short-latency (presumably monosynaptic: ‘direct’) and long-latency (presumably polysynaptic: ‘re-entrant’) responses were observed. Facilitation of direct responses was modest in 3xTgAD animals. However, re-entrant responses in DG and CA1 of young 3xTgAD mice developed earlier in the stimulus train and with larger amplitude when compared to controls. Old mice showed less DG paired-pulse depression and no evidence for re-entrance. In summary, DG and CA1 responses to low-frequency stimulation in all groups were comparable, suggesting no loss of synaptic connectivity in 3xTgAD mice. However, higher-frequency activation revealed complex change in synaptic excitability in DG and CA1 of 3xTgAD mice. In particular, short-term plasticity in DG and CA1 was facilitated in 3xTgAD mice, most evidently in younger animals. In addition, re-entrance was facilitated in young 3xTgAD mice. Overall, these data suggest that the episodic-like memory deficit in 3xTgAD mice could be due to the development of an abnormal hyper-excitable state in the hippocampal formation.

## Introduction

Alzheimer's disease (AD) is characterised phenotypically by profound declarative memory deficits. The hippocampus, vital for both the formation and retrieval of declarative memory, is one of the first areas affected by AD pathological hallmarks of amyloid-beta (Aβ), extracellular plaques and tau neurofibrillary tangles [1], [2]. Interestingly, however, overt plaque burden correlates poorly with cognitive decline in AD patients [3]. A more recent hypothesis is that intracellular oligomeric amyloid species, present before the accumulation of extracellular plaque pathology, may instead play a pivotal role in disease progression [4], [5]. However, despite extensive research examining the causes of AD pathology and characterising behavioural phenotypes, there is still little knowledge about the physiological basis for memory loss in pathological states such as AD [6].

The hippocampal formation is comprised of the dentate gyrus (DG), hippocampus proper (CA fields), subiculum (SUB), parasubiculum, presubiculum and entorhinal cortex (EC; medial and lateral divisions; MEC and LEC). CA1 and subiculum are the principal output structures of the hippocampus [7] and EC provides the interface between incoming and outgoing information from surrounding cortex [8]. Hippocampal output can return to surrounding neocortex via deep layers of EC and/or re-enter the hippocampal formation via projections from deep to superficial EC [9], [10]. The latter reverberation (or ‘re-entrance’) is considered to be pivotal in memory formation, possibly acting as a comparator mechanism to allow processed input to be evaluated alongside new information and/or as a memory consolidation mechanism during sleep [7], [11]–[13]. Selective lesions to all hippocampal formation structures have profound effects on memory performance [14]–[18]. Functional abnormalities have also been detected in the hippocampus during memory encoding in human AD patients [19]. It is of major interest, therefore, to determine the pathophysiological profile of the hippocampal formation in AD models and to elucidate, in particular, whether reverberation (re-entrance) occurs in control and AD model mice.

The triple transgenic mouse model (3xTgAD) carries familial AD transgenes for Amyloid Precursor Protein (APP_SWE_), Presenilin-1(M146V) and an additional tauopathy mutation (Tau_P301L_). This model develops Aβ and tau pathology targeted to the hippocampus and other medial temporal lobe structures in a manner temporally and spatially similar to human AD [20], [21]. The model develops cognitive deficits at a young age [21], [22] and, as we have demonstrated recently, a specific decline in episodic-like memory from 3 months that becomes a complete deficit at 6 months of age [23], [24]. 3xTgAD mice also show abnormalities in basal synaptic transmission and deficits in LTP in CA1 hippocampus *in vitro* [21]. However, to date, there have been no electrophysiological recordings *in vivo* in the 3xTgAD mouse and, to our knowledge, there is only one *in vivo* account in another AD model examining evoked responses [25]. Thus, the hippocampal system in AD mouse models has largely been examined only in the reduced brain slice preparation [21], [26], that is, without the substantial bi-directional cortical connectivity that also contributes to memory processes [27]. In the 3xTgAD mouse, only CA3-CA1 connectivity has been examined directly *in vitro* [21], therefore, there is a need to examine basic synaptic transmission more widely in hippocampal formation and also hippocampal network function through examining response reverberation.

One question that arises is the extent to which cognitive deficits seen in early AD stem from abnormalities in the fibre pathways projecting to the hippocampus and/or within hippocampal subfields. Recent research has indicated the presence of early myelination abnormalities in the 3xTgAD mouse Schaffer collateral pathway, prior to the occurrence of Aβ and tau pathology [28]. In human AD, tau pathology is restricted to the stellate cells in EC layer II, and leads to degenerative changes in the perforant path [29]. In addition, afferents from CA1 and SUB to EC are major sites for Aβ pathology in human AD [16]; thus, there is a strong possibility that information flow through the hippocampal formation could become impaired in AD. However, synaptic deficits first arise in AD models *in vitro* at an age prior to overt extracellular plaque and tangle pathology, suggesting that mechanisms such as intracellular Aβ accumulation or abnormalities in calcium homeostasis are major contributing factors to early cognitive symptoms [21], [26].

Here, we examined the functional state of the hippocampal formation in the 3xTgAD mouse through the recording of extracellular field potentials *in vivo*. Whereas most similar studies focus on one region of hippocampus in isolation (e.g., CA1 or DG), in this study we measured synaptic integrity and short-term plasticity at three sites simultaneously, specifically, the granule cell layer of DG, CA1 stratum radiatum (CA1sr) and CA1 stratum lacunosum-moleculare (CA1slm), using a multi-site electrode recording approach [9]. These sites were chosen as regions that receive monosynaptic input from the perforant path and, for CA1sr, polysynaptic feed forward input from DG via CA3. We measured extracellular field excitatory post-synaptic potentials (fEPSPs) in these target regions and performed current-source-density (CSD) analyses on CA1-DG axis responses. Through this we aimed to map synaptic current flow, indicative of synaptic-activity throughout the laminar structure, to test the integrity of synaptic connections within the EC-hippocampal tri-synaptic circuit.

Electrophysiological recordings conducted in AD mouse lines *in vitro* support a long-term decrease in synaptic function [21], [30]–[32]. However, *in vivo* recordings support the presence of hyper-excitability and epileptiform activity, at least in early pathological stages, which are thought to contribute to cognitive decline [33], [34]. In addition, recent findings indicate the presence of spontaneous seizure activity in the 3xTgAD model [35]. Thus, in the current study, we hoped to further examine these contradictory reports of decreased/increased synaptic function and test whether our observed decline in episodic-like memory in the 3xTgAD model [23], [24] correlates with excitability changes within hippocampal circuits *in vivo*.

In contrast to *in vitro* reports, we found little evidence for decreased functional connectivity in either young or old 3xTgAD mice. Indeed, in measures of short-term plasticity we saw evidence for increased DG and CA1sr excitability in 3xTgAD mice compared to controls, particularly in young mice. This was coupled, however, with relatively less facilitated fEPSP amplitudes to a stimulus train alongside an enhanced re-entrant response into the hippocampal circuit in 3xTgAD mice. Thus, our data support increased hippocampal excitability at two loci in the 3xTgAD mouse: firstly, EC layer II input to DG and CA1sr (the latter presumably via propagation of DG output via CA3) in young and aged mice; and secondly, facilitation of hippocampal re-entrance in young 3xTgAD animals, presumably through potentiated deep-to-superficial EC connectivity.

## Materials and Methods

### Animals

Triple-transgenic mice (3xTgAD) carrying APP_SWE_, PS1_M146V_ and Tau_P301L_ transgenes and matched controls were bred at the University of Manchester from a colony donated by the La Ferla group [21]. Mice were housed in same-sex and genotype groups of 5–6 individuals on a 12∶12 light/dark cycle with access to food and water *ad libitum*. The 3xTgAD colony was maintained through the pairing of homozygous individuals and the presence of transgenes was confirmed by genotyping a subset of 3xTgAD mice in each cage. All procedures conformed to UK Home Office licensing (project license PPL 40/3231) and were approved by the University of Manchester Ethical Review Panel. Electrophysiological data were collected from a ‘young’ sample of mice aged 4–6 months comprising 12 control (6 males: 6 female) and 11 3xTgAD (5 males: 6 female) mice. The age of the young group was selected to coincide with the early presence of hippocampal intracellular Aβ pathology (plaques and tangles accumulate from approximately 9 months of age in 3xTgAD mice) and hippocampal-dependent memory deficits [21]–[24]. A second ‘old’ sample of mice at 17–18 months of age comprised 5 control and 4 3xTgAD mice (all female); at this age there is extracellular plaque and tau pathology within hippocampus and subiculum. Thus, these groups allowed us to explore the additive effect of both pathology and general ageing on DG and CA1 responsiveness [20]–[22]. All female mice had previously experienced a battery of behavioural testing (spontaneous recognition tasks) which ceased at least a month before electrophysiological recordings were conducted. Male mice used for electrophysiology were experimentally naive. In the present experiments data from young male and female mice were combined as no sex differences were seen in either their basal input/output response, or in their CSD profiles to a 5 Hz train (data not shown).

### Surgery and equipment

**Figure 1:**
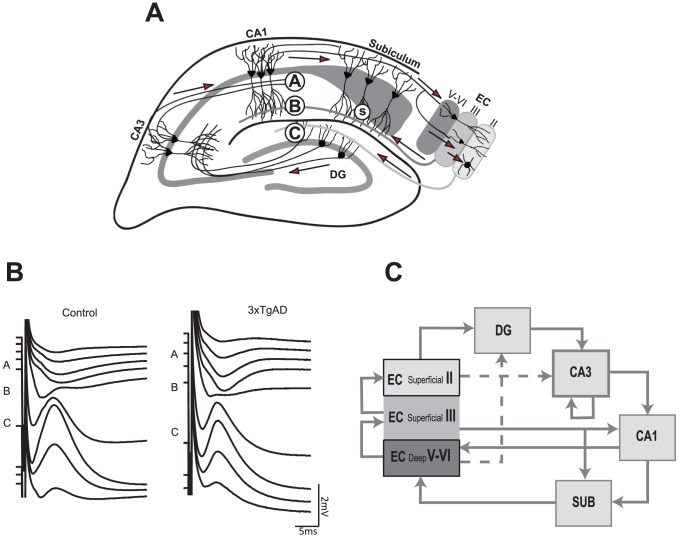
The hippocampal tri-synaptic circuit. A: Hippocampal formation with approximate electrode placements marked. Input and output pathways with principal direction of synaptic flow (arrows) and termination of fibre pathways from Schaffer collaterals [A], PP layer III [B] and PP layer II [C] are marked. Evoked activity at these points corresponds to CA1sr [A], CA1slm [B] and DG responses [C]. Stimulating electrode placement was in the deep dendritic layer of subiculum (S). B: Typical laminar field response to single-pulse stimulation at half-maximal current intensity. A, B and C correspond to responses recorded within regions A–F in Figure 3. C: Block diagram of the connectivity displayed in [A] for reader's clarity. Dashed lines represent the presence of sparse regional connectivity. Calibration applies to both profiles in B.

Mice were anaesthetised (urethane 30% w/v in dH_2_O, 1.6 g/kg, i.p) and monitored until areflexia was achieved. Where reflexes remained, a small additional dose of urethane (10% w/v in dH_2_O, 20–30 µl, i.p.) was given. The mouse was held in a stereotaxic frame (Kopf, 1430, USA) with lamda and bregma in the same horizontal plane to match the mouse atlas of Paxinos and Franklin [36]. A homoeothermic heating blanket (Harvard Apparatus, UK) and rectal probe maintained core body temperature at 37°C. A midline scalp incision was made and the skull exposed. Craniotomies were drilled above CA1 (B–2 mm, ML 1.5 mm) for the recording electrode and dorsal subiculum (B-3.85 mm, ML 1.5 mm) for the stimulating electrode. Recordings were made using a linear multi-electrode recording array containing either 16 contacts spaced100 µm, or 32 contacts spaced 50 µm apart (both 413 µm^2^ contact area; NeuroNexus Tech, USA; there were no clear differences in response profiles between the different contact arrangements so data from different arrays were pooled.). The array was lowered vertically into the brain until the tip was approximately 2.5 mm below brain surface; at this depth recording contacts spanned the CA1-DG axis fully ([9]; see Figure 1A). A bipolar stimulating electrode (twisted 125 µm diameter Teflon-insulated stainless-steel wires; Advent RM, UK) was inserted 1.5 mm from brain surface at 30° relative to vertical into the molecular layer of the mid antero-posterior part of the subiculum (Figure 1A). At this position, stimulus pulses would activate the hippocampus both directly (via activation of perforant path (PP) fibres traversing subiculum) and indirectly (via re-entrance through subicular output to deep layers of entorhinal cortex which then activate superficial entorhinal PP output [9], [10]. It was expected that upon stimulation, a characteristic pattern of current sources and sinks would be identifiable across the CA1-DG axis similar to those seen in the rat, reflecting the termination pattern of PP inputs to DG and CA1 ([9]; see Figure 1B for typical evoked laminar response and C for hippocampal circuit diagram).

### Stimulation Protocols

Stimulus pulses were timed using a National Instruments card (PCI-6071E, NI, UK) controlled using LabVIEW software (8.2, NI, UK). These triggered a constant-current source (DS3, Digitimer, UK) connected to the stimulating electrode. Stimulus duration was set to 0.2 ms throughout the experiments. Stimulus protocols consisted of single pulses (0.33 Hz), paired pulses (PPulse) and trains of 20 pulses.

We first confirmed that the recording electrode spanned CA1sr, CA1slm and the superior and inferior blades of DG. This was achieved by monitoring the presence of stable evoked local field potentials (LFPs) in these regions to single-pulse stimulation at 200 µA [9], [10]. In all experiments, a characteristic DG molecular layer positive-going LFP component was recorded with, in most cases, a granule cell layer population spike (PS; latter visible with high intensity PPulse or train stimulation; Figure 1B point C). The CA1slm response (presumably from activation of direct EC layer III input) was not seen in all experiments, likely due to variations in the placement of recording/stimulating electrodes (Figure 1B point B). CA1sr was characterised by a longer-latency negative-going LFP (Figure 1B point A). Due to the fixed distance between contacts in the recording array, upon identification of a typical response in one layer, those in other layers could reliably be confirmed due to the distance between contacts and LFP profile (CA1sr 100 microns dorsal to CA1slm, CA1slm approximately 100 microns dorsal to the largest DG molecular layer response; see Figure 1B for illustration of spacing).

Once a characteristic laminar LFP profile was achieved, a current-voltage (input-output) response curve was recorded for each LFP component by applying pairs of pulses at 50 ms intervals over a range of current intensities (50–600 µA). The first pulse of each pair was analysed and used to plot the curve. Thereafter, for PPulse and train protocols, the stimulus current was set to the value required to evoke a half-maximal response (i.e., 50% of the value required to elicit the maximum fEPSP in CA1sr); this was typically in the range 100–200 µA.

To examine short-term plasticity, PPulses were delivered at inter-pulse-intervals (PPI) of 25, 50, 100, 200, 500 and 1000 ms. All pairs were separated by 3 seconds and repeated 20 times. To examine the effects of repetitive stimulation and re-entrance into the hippocampal circuit, a single train of 20 pulses was applied at a frequency of 5 Hz. This frequency was chosen to provide a comparison with PPulse intervals and we predicted that 5 Hz trains would not produce any long-term plastic changes [9], [10].

Upon completion of all stimulation protocols, lesions were created to mark electrode placements displaying particular response components [37]. Mice were perfused transcardially with 0.2 M sodium phosphate buffer and 4% paraformaldehyde and brains removed for fixation. Electrode placements were confirmed from 30 µm thick, Nissl-stained sagittal brain sections.

### Data Acquisition and Analysis

Signals were amplified at source through an AC-coupled headstage (x20 gain) and further amplified for a total gain of 500x (Recorder64 system, Plexon, USA). Signals were filtered (0.1 Hz–6 kHz) and sampled at 10 kHz per channel (12-bit resolution).

Raw fEPSP response amplitudes to single-pulse stimulation were plotted to describe the current-response relationship (input-output; I/O) in control and 3xTgAD mice. In addition, PPulse and train data were normalised to the first pulse and results shown as a percentage change for both amplitude and latency to peak/trough to demonstrate any facilitation or depression of responses. Finally, train data were subjected to 1D CSD analysis [38] using custom Matlab software (see below).

All numerical data were analysed using Prism (v5; Graphpad). Mixed ANOVA with Bonferroni post hoc comparisons were applied for identification of pair-wise genotype differences for each of the three measures (I/O, PPulse and trains) for each response (CA1sr, CA1slm and DG). Due to sex differences in the number of animals present in each sample, young and old data sets were analysed separately to control for the small female-only older mouse group; however, young male and female data were then pooled as no sex difference was seen for any measure (data not shown). If recordings became unstable during an experiment, stimulation protocol data were excluded for that animal. Final sample sizes for each hippocampal layer in each stimulation protocol are shown in Table 1.

**Table 1:** Summary of data included in statistical analyses.

Young (4–6 months)						
	Input/Output		Paired Pulse		Trains	
	Control	3xTgAD	Control	3xTgAD	Control	3xTgAD
CA1sr	n = 12	n = 11	n = 12	n = 10	n = 10	n = 9
CA1slm	n = 12	n = 11	n = 12	n = 10	n = 7	n = 8
DG	n = 12	n = 11	n = 12	n = 10	n = 11	n = 9

Old (17–18 months)						
	Input/Output		Paired Pulse		Trains	
	Control	3xTgAD	Control	3xTgAD	Control	3xTgAD
CA1sr	n = 5	n = 4	n = 5	n = 4	n = 5	n = 3
CA1slm	n = 5	n = 4	n = 5	n = 4	n = 5	n = 3
DG	n = 5	n = 4	n = 5	n = 4	n = 5	n = 3

### Amplitude, Slope and Latency measurements

Amplitude and the latency of evoked responses were extracted through custom Matlab software (Mathworks, version 7.0). In brief, each response was first visually inspected to ensure it had the expected shape with no noise or other artefacts. Five cursor points were then marked on different components of a mean response (see below) averaged over the 20 stimulus-response repeats. Thereafter, the programme used this five-point template to calculate the amplitude and latency of each original response. For train data, the waveform of each pulse was measured ‘by hand’ to account for the rapid changes in response profile associated with repetitive stimulation. In both cases, five points on each response were marked as follows: point 1 as the sample point before the start of the stimulus artefact; points 2 and 3 surrounded the response onset and points 5 and 6 surrounded the response peak (or trough).

For response amplitude (mV), the mean value between points 2–3 was subtracted from the corresponding maximal (response peak) or minimal (response trough) value between points 4 and 5. For latency to response peak/trough (ms), the centre timestamp between points 4 and 5 was subtracted from that for point 1. We did not attempt to measure latency to response onset, as there was seldom a defined level period in the recording between the stimulus artefact and response. All data presented in this paper are the average of 20 pulses. For DG population spikes (PS), the probability of evoking a PS was calculated out of 20 sweeps for each PPI in animals that demonstrated PS at half-maximum stimulation.

### Current Source Density Analysis

Current Source Density (CSD) analysis when applied to extracellular recordings taken from a laminar structure provides a highly detailed, anatomically aligned, spatiotemporal map of current sinks and sources produced through synaptic activity.

Single responses from train data were analysed by one-dimensional CSD analysis in a custom built Matlab programme, estimating the second-order spatial derivative using the formula of Freeman and Nicholson [38]. For this the distance between electrodes was either 100 or 50 µm and the degree of spatial smoothing applied was 2 as we analysed neighbouring contacts. It was assumed that (a) the major extracellular currents ran in parallel to the recording electrode (along the CA1-DG axis) and (b) tissue conductivity was spatially homogenous across the recording array (that is, any residual currents would represent local synaptic sinks and sources). CSD values at locations between those calculated for each electrode position were estimated by linear interpolation (20 steps) to produce a smooth and continuous mapped depiction of synaptic currents. Mapped CSD values are presented here in arbitrary units: current sources were mapped as reds, oranges and yellows; current neutral regions in light green; and regions showing current sinks as dark green to dark blue. The colour map for each CSD plot was normalised to itself (max/min) to represent the current gradient running from its maximum positive current (hot colours), to the maximum negative current (cool colours) with green equivalent to resting state or neutral.

## Results

### Current-response relationship

**Figure 2:**
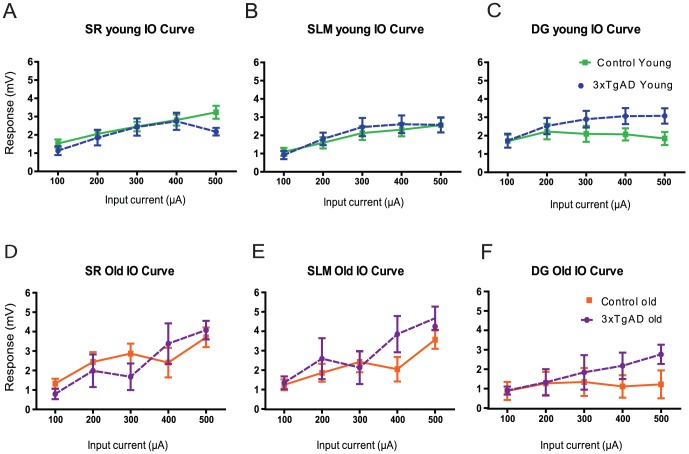
Input-output relationships in young and old 3xTgAD mice are similar to controls. A to F: Stimulus-response (I/O) curves in young (4–6 month) and old (17–18 months) animals in relation to increasing input current intensity at CA1sr (A and D), CA1slm (B and E) and DG (C and F). Error bars are (±SEM).

To examine the integrity of CA1 sr/slm-DG hippocampal circuitry, amplitude and latency of responses to activation of PP fibres were determined over a range of current intensities. There were no significant differences in amplitude measurements between control and 3xTgAD animals for CA1sr, CA1slm or DG responses in either young or old mice (Figure 2A–F; genotype (2) by PPI (5) mixed ANOVA). However, there was a significant effect of current intensity on all response amplitudes in all animals (F (4,28)  = 5.74–23.06 P<0.0001), with higher currents eliciting larger responses as would be expected by recruitment of PP fibres with increasing current. In addition, DG population spikes developed from 200 µA stimulus current onwards in 5/12 control and 8/11 3xTgAD young mice (data not shown). In old mice, two of the four 3xTgAD animals developed DG PS at high current intensities; however, PS were not seen in old control mice (data not shown). While these observations in DG PS frequency are merely qualitative for the purpose responses to single stimuli, their occurrence was strain-dependent in 3xTgADs and controls during PPulse stimulation, as described below.

To investigate differences in response latency between 3xTgAd and control mice we compared responses from each hippocampal layer at a stimulus intensity of 200 µA (typical half-maximal stimulation current; see Table 2). The order of latencies (to response peak or trough) was consistent with our expectations for the associated synaptic delays *in vivo* of CA1sr, CA1slm and DG in response to PP stimulation as used here (i.e., fastest for DG and CA1slm; refer to Figure 1C) and no genotype differences were found (data not shown). Thus, from the similar I/O amplitude and relative response latency patterns seen with this stimulus protocol, we concluded that there were no genotype differences in functional connectivity during low-frequency activation in the PP fibre pathways from EC layers II and III traversing subiculum to the hippocampal circuit in both young and old animals.

**Table 2:** Response latencies in hippocampal formation.

	Control 4–6 mo			3xTgAD 4–6 mo			Control 17–18 mo			3xTgAD 17–18 mo		
	Latency	SEM	n	Latency	SEM	n	Latency	SEM	n	Latency	SEM	n
**CA1sr**	7.84	0.56	12	7.99	0.54	11	9.79	1.21	5	9.25	1.64	4
**CA1slm**	6.01	0.49	12	5.26	0.42	11	7.30	1.21	5	6.73	0.82	4
**DG**	8.03	0.65	12	7.46	0.36	11	6.69	2.24	5	7.81	1.26	4

Figures show peak response latencies (ms) in response to subiculum stimulation at 200 µA.

### Short-term synaptic plasticity

To assess short-term synaptic plasticity, we examined the effect of varying PPI on the amplitude and latency of responses to half-maximal stimulus current (range for all mice 100–200 µA). Measurements from the second pulse were normalised to those from the first and are presented as percentage change, such that positive values represent paired-pulse facilitation (or a decreased latency) and negative values represent paired-pulse depression (or an increased latency). CA1sr, CA1slm and DG responses were analysed in separate genotype (2) by PPI (6) mixed ANOVA with Bonferroni post-hoc comparisons.

**Figure 3:**
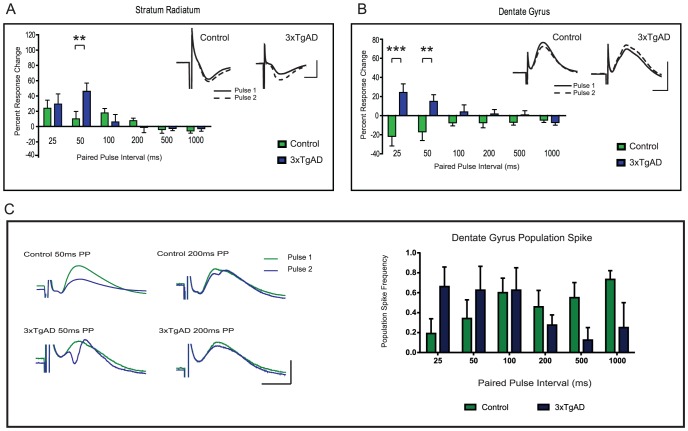
Increased short-term plasticity in young 3xTgAD mice. Panels A and B show fEPSP amplitude of pulse 2 normalised to pulse 1 (±SEM) for CA1sr [A], and DG [B]. No differences were observed for CA1slm (data not shown). Inserts show representative traces from single experiments with pulse 1 (black line) and pulse 2 (grey line) taken from PP50ms in all examples. Pair-wise genotype differences are evident (**P<0.01; ***P<0.0001). Calibration 12.5 ms/2 mV. C: Left panel shows examples of population spikes (PS) evoked at 50 or 200 ms in control and 3xTgAD mice (average of 20 sweeps each; calibration 10 ms/3 mV). Right panel shows the probability of evoking a PS to the second pulse of a stimulus pair versus paired-pulse interval. Note that the PS probability is highest at short intervals for 3xTgAD and longer intervals for controls. Error bars are (±SEM).

For young animals, there was a significant effect of PPI on the amplitude of CA1sr and CA1slm responses (F(5,100) = 7.32, P<0.0001 and F(5,100) = 4.93, P<0.0005, respectively) with maximal facilitation seen at 25 and 50 ms intervals. While there were no significant genotype differences for CA1slm responses (data not shown), for CA1sr there was an interaction (F(5,100) = 2.54, P<0.05) and a pair-wise genotype difference at 50 ms (t(20) = 3.27, P<0.01; see Figure 3A). This was evident as 3xTgAD responses facilitating significantly more than that for controls, in stark contrast to the depression of CA1sr responses frequently seen in AD models *in vitro* during LTP protocols [21], [31], [32]. For the DG response, there was again a significant genotype difference (F (1,100)  = 11.80, P<0.005) and an interaction (F(5,100)  = 4.88, P<0.001). Furthermore, there were pair-wise genotype differences at 25 ms (t(20) = 5.12, P<0.001) and 50 ms (t(20) = 3.54, P<0.01), where response amplitudes facilitated in 3xTgAD mice and depressed in controls (Figure 3B), suggestive of DG hyper-excitability in this AD model. As expected, population spikes to the second pulse (P2) were seen in the DG granule cell layer in 4/10 3xTgAD and 6/12 controls during half-maximal amplitude PP stimulation. However, 3xTgAD mice had a higher tendency to exhibit PS at shorter PPI (25 ms–100 ms), whereas for control animals this relationship was reversed (most PS in 200 ms–1000 ms PP interval range; examples of paired-pulse responses at 50 ms and 200 ms PPI left panel Figure 3C, probability distribution across PPI for PS to P2 displayed in right panel Figure 3C).

To determine whether genotype differences in the velocity of synaptic transmission could have contributed to observed short-term amplitude differences, response latencies were examined for each hippocampal layer in response to pairs of pulses (latency of second pulse to peak/trough relative to the first). There was a significant effect of PPI on latency for CA1sr (F(5,100)  = 2.63, P<0.05) with PPI 25 ms eliciting a slightly faster response for both genotypes (3xTgAD 14.99%±3.17 SEM faster and control 5.82%±1.7 SEM faster than the first pulse). For DG, a significant effect of PPI was also seen (F(5,100) = 7.41,P<0.001), with PPI 100 ms and 200 ms eliciting a slightly slower response in both genotypes (data not shown); however, no effects of latency were observed in CA1slm. Thus, despite a significant effect of PPI on the time to peak/trough of the second pulse, there were no obvious effects of AD pathology. To summarise the data from young animals, we found an indication of increased short-term excitability in 3xTgAD mice in the form of increased LFP amplitude in CA1sr, and with DG facilitation to short interval delays; however, there was no apparent change in overt hippocampal synaptic connectivity as reflected in the absolute latencies of CA1sr and DG molecular layer LFPs.

**Figure 4:**
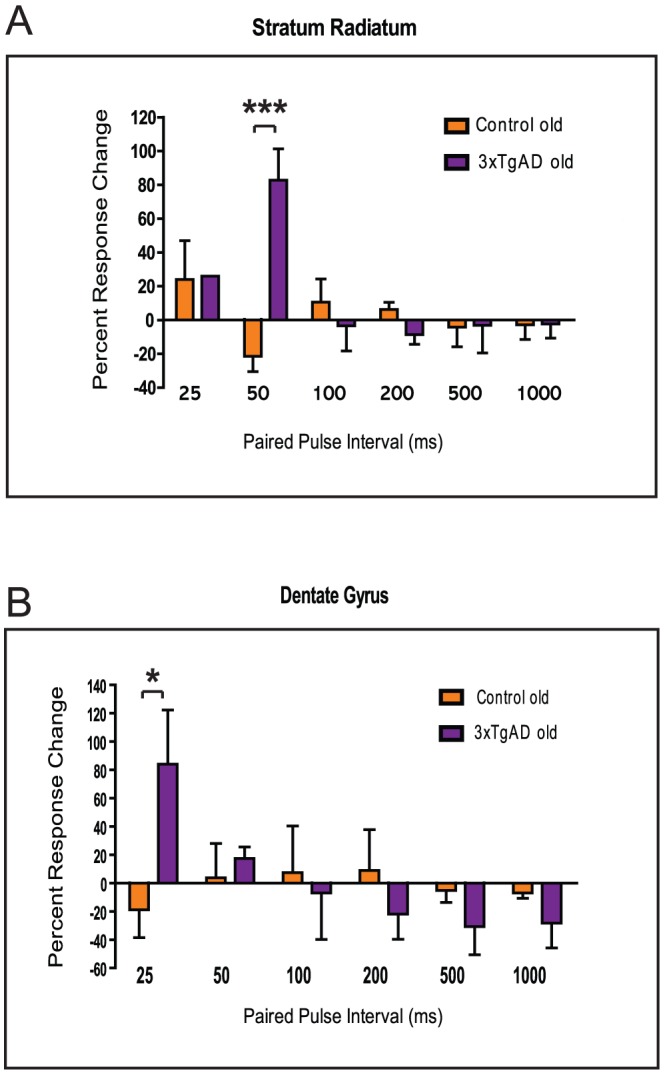
Increased short-term plasticity in old 3xTgAD mice. Panels A and B as per Fig 3 for CA1sr [A] and DG [B]. Pairwise genotype differences are *P<0.05; ***P<0.0001. Note lack of modulatory effect of PPI in controls for DG.

For old animals there was a significant effect of PPI on response amplitude for CA1sr (F(5,35) = 2.54, P<0.05) and a significant interaction (F(5,35) = 5.94, P<0.0005). This manifested as a significant pair-wise genotype difference at PPI 50 ms (t(7)  = 5.65,P<0.0001; Figure 4A) where 3xTgAD animals again facilitated more than controls. There were no significant effects for CA1slm; however, there was an interaction for DG (F(5,35)  = 3.46,P<0.05) and a pair-wise genotype difference at PPI 25 ms (t(7)  = 3.08), P<0.05; Figure 4B), where again 3xTgAD responses facilitated in contrast to control responses which depressed. Of note, 3xTgAD DG responses tended to depress more strongly than controls at PPI of 200 ms or longer, however, these differences did not reach significance. Also of interest, control DG appeared to show little if any modulatory changes across the range of PPIs, neither depressing nor facilitating (compare Figure 3B with Figure 4B). We assume these were age-related changes in DG function, as they present a different profile to that of aged 3xTgAD animals. The implication of this reduction in paired-pulse depression for DG with normal ageing is that it may be causative in the known age-related impairment in pattern separation that may, in turn, contribute to our observed age-related deficit in episodic-like memory in older control mice ([23]; see Discussion).

For latency to peak/trough, there was a significant effect of PPI for CA1sr (F(5,35) = 2.69, P<0.05), an interaction (F(5,35) = 4.23, P<0.005) and a pair-wise genotype difference at PPI 50 ms (t(7) = 3.99, P<0.05). In this case, 3xTgAD mice had a significantly longer latency relative to P1, whereas control mice had a slightly shorter latency (3xTgAD change 59.42% ±27.83 SEM slower, control 8.08%±3.49 SEM faster). This difference can be explained by the larger CA1sr amplitudes in 3xTgAD mice compared to controls (3xTgAD responses have a longer period from stimulus to response trough). For CA1slm, there was a significant interaction (F(5,35)  = 4.35, P<0.01) and a pair-wise genotype difference at PPI 50 ms (t(7) = 3.01,P<0.05) with 3xTgAD mice showing a faster P2 response than control mice (3xTgAD 10.79%±4.28 SEM faster, control 1.47%±5.26 SEM slower). For DG, there was an effect of PPI only (F(5,35)  = 3.02, P<0.05). In these old animals, only one 3xTgAD mouse showed evidence of a DG PS and, as per the young animals, this was more prevalent at shorter PPI (PP25ms and PP50ms; data not shown). Thus, as per the young data, it appeared that old 3xTgAD mice demonstrate increased short-term plasticity and excitability in CA1sr and DG, albeit to a smaller degree when compared to younger mice.

To summarise the findings of the PPulse protocol, both young and old animals showed response facilitation to PPulse stimulation at short intervals in CA1sr; however, excitability in terms of increases in DG PS to short PPI was most pronounced at a young age. 3xTgAD mice had a significantly larger CA1sr response than controls and this was seen in both young and old age groups, suggesting a specific change at the CA1 level could alter excitability in this region. In addition, 3xTgAD DG responses at a short PPI were facilitated relative to controls (which depressed in young controls), indicating changes in the DG synaptic network. The DG paired-pulse depression in young controls appeared to be absent in old controls, suggesting an ageing-related shift in DG function; this attenuation may have mechanistic implications for age-related deficits in the pattern separation role carried out by DG and episodic-like memory capacity in older mice.

### Train stimulation

To examine neuronal reverberation within the hippocampal formation, mice experienced low-frequency stimulation in the form of a 20-pulse train, delivered at 5 Hz. Train data were analysed in genotype (2) by pulse number (20) Mixed ANOVA as follows: raw amplitudes and peak/trough latencies were examined for each response (results labelled as ‘raw’) and data were also converted to a percentage change from P1, as a measure of the summated effect of train stimulation (labelled as ‘% change’). Data from young and old mice were also subjected to CSD analyses to identify the spatiotemporal relationships between current sources and sinks along the CA1-DG axis and to shed light on the origins and relative strength of the synaptic inputs to different laminae.

### 5 Hz Train analysis: Young animals at 4–6 months of age

For young animals, there was a significant effect of pulse number on the amplitude for all responses; however, for latency, there was a significant effect of pulse number only for CA1slm and DG responses and a genotype difference for CA1sr.

**Figure 5:**
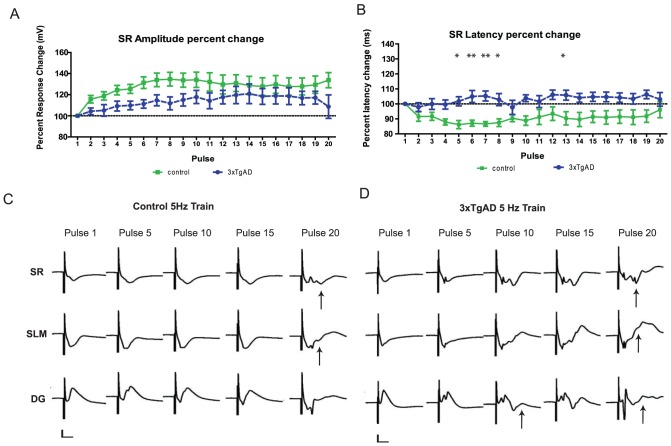
Changes in response latency and occurrence of hippocampal re-entrance during 5 Hz stimulus trains in young 3xTgAD animals. A & B: fEPSP amplitude [A] and latency [B] for CA1sr response, expressed as a percentage change from the response to pulse 1. Pair-wise genotype differences at *P<0.05 and **P<0.01. C & D: Example to show differential fEPSP responses to selected stimuli of a 5 Hz train in control [C] and 3xTgAD [D] mice classified as ‘excitable’. Responses in ‘non-excitable’ animals during 5 Hz trains remained as per the response to pulse 1. Re-entrance (longer latency responses, likely propagating through EC) are shown with upward pointing black arrows for pulse 20 in both genotypes and, in this instance, by pulse 10 in [D]. Calibration bar [C,D] 2 mV by 10 ms.

CA1sr (n = 10 control, n = 9 3xTgAD) showed a significant effect of pulse number on amplitude (F(19, 323) = 5.94, P<0.0001 raw; F(19,323) = 6.18, P<0.0001%change; Figure 5A), that is, subsequent pulses facilitated with respect to pulse 1. However, despite the tendency for 3xTgAD mice to show less facilitation throughout the train this genotype difference did not reach significance. There was also no effect of pulse number on response latency, however, there was a significant genotype difference (F(1,323) = 11.01, P<0.005%change) and an interaction (F(19,323) = 1.63, P<0.05), with Bonferroni pair-wise differences at pulses 5–8 and 13 (t(17) = 3.071–3.725, P<0.05/P<0.01; Figure 5B). In this case, 3xTgAD response latencies remained the same or were slightly shorter compared to that of the first pulse, whereas control latencies increased.

CA1slm response amplitudes (n = 7 control, n = 8 3xTgAD) showed a significant effect of pulse number only (F(19, 247) = 5.96, P<0.0001 raw; F(19, 247) = 6.07, P<0.0001, %change) as did CA1slm latencies (F(19,247)  = 3.37, P<0.0001 raw; F(19, 247) = 1.84, P<0.05%change) with progressive pulses evoking a larger and slightly longer latency response (data not shown). There were no genotype differences.

DG response amplitudes (n = 11 control, n = 9 3xTgAD) showed a significant effect of pulse number (F(19,342) = 3.5, P<0.0001 raw; F(19,343) = 10.36, P<0.0001%change) as did response latencies (F(19, 342) = 3.69, P<0.0001 raw; F(19, 342) = 8, P<0.0001), with subsequent responses depressing slightly and latencies increasing compared to the first response. There were no genotype differences for either response amplitude or latency (data not shown).

In summary, there was a robust effect of pulse number on both the amplitude and latencies of responses. We found no significant evidence for genotype differences in the absolute amplitude of CA1 and DG responses; however, CA1sr clearly demonstrated a trend for poorer facilitation in 3xTgAD animals with an accompanying genotype difference in CA1sr latency in 3xTgAD mice.

### Re-entrance into CA1 and DG: young mice

Half of the young mice from each genotype were classified as ‘excitable’ (5/11 control and 5/9 3xTgAD), that is, they showed increasing levels of DG population spiking for subsequent pulses in the train and, in some cases, re-entrance into the hippocampal circuit in both CA1 and DG (Figure 5 panels C and D; see arrows). This is better illustrated using 1-dimensional CSD analyses, where the evoked synaptic currents in each layer of the hippocampus can be computed and visualised over time (see next section). All animals that showed re-entrance did so by pulse 20: within the 5 excitable 3xTgAD animals, 3 showed clear re-entrance into CA1sr (at pulses 10, 17 and 20; 15.71±0.33 ms; mean latency ± SEM), 4 showed re-entrance into CA1slm (at pluses 15, 18 and twice by 20; 14.71±0.12 ms) and 3 exhibited re-entrance into DG (at pulses 7, 15 and 20 pulses; 20.69±1.98; Figure 5D). Re-entrance latencies were similar in the 5 excitable control mice; CA1sr (n = 4; 17.5±1.5 ms) and CA1slm (n = 3; 15.49±2.08 ms), whereas DG had a shorter latency re-entrance wave (n = 2; 16.32±2.22 ms). However, in contrast to 3xTgAD mice, all re-entrance was seen at pulse 20 of the train and not earlier in the sequence. It appears, therefore, that young control and 3xTgAD mice (of 4–6 months of age) can show self-sustained reverberation through the hippocampal formation following repetitive low-frequency stimulation; in addition, these responses tend to occur in 3xTgAD mice at an earlier stage in the pulse sequence.

### Current Source Density analysis: young mice

**Figure 6:**
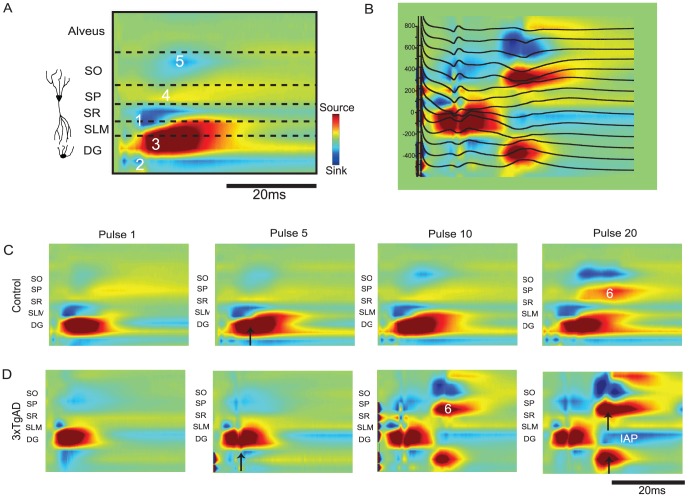
Laminar CSD profiles for responses to 5 A: CSD from pulse 10 of control mouse with approximate layer boundaries indicated (SO-stratum oriens, SP-stratum pyramidale, SR-stratum radiatum, SLM-stratum lacunosum-moleculare and DG-Dentate gyrus and Alveus). Left side of all CSD panels represents point of stimulus onset. Current sources are yellow/red, current sinks light/dark blues and neutral regions are in green. The overlaid numbers 1–6 seen in [A] and in [C–D] correspond to synaptic events as follows: slm sink (1), DG molecular sink (2), DG granule cell source (3), CA1 source (4), SO sink (5) and CA1 long latency source (6). B: 3xTgAD CSD laminar profile with voltage overlay of evoked response corresponding to pulse 20 in [D]. Long-latency re-entrance and PS can be seen in CA1. CSD figures from control [C] and 3xTgAD [D] mice correspond to the field response profiles for the pulses seen in Figure 5C and D. CSD analysis of pulses 1, 5, 10, and 20 of a 20-pulse train shows progressive increases in excitability in CA1 and DG cell layers in control [C] and 3xTgAD [D] mouse. Black arrows point to short-early DG PS (pulse 5), inferior blade DG PS (pulse 20 3xTgAD) and long-latency CA1 PS that appears to propagate away from CA1 through the alveus (pulse 20 3xTgAD). Presumed inhibitory-afterpotentials labelled as IAP. Scale bar is 20 ms. CSD figures are scaled to the same parameters for each pulse and between genotypes.

Stimulation of the PP with a 5 Hz frequency train elicited a profile of current sources and sinks consistent with an electrode traversing the CA1-DG axis, with positioning parallel to the dendrites of pyramidal cells in CA1 and spanning DG [9]. Typical CSD responses in and their layers of origin are outlined in Figure 6A (control mouse response to the 5^th^ pulse).

The CSD responses for control and 3xTgAD animals can be viewed for pulses 1, 5, 10 and 20 in Figure 6 C and D. To orientate the reader to the appearance of each synaptic component, the CSD responses to pulse 1 are referred to using the example in Figure 6A. In response to pulse 1, both 3xTgAD and control animals show an early latency current sink in CA1slm (Figure 6A: component 1) consistent with an excitatory synaptic response to activation of perforant path (EC layer III tempero-ammonic) terminals on the apical dendrites of CA1 pyramidal cells. Simultaneous with this, a sink in the molecular layer of the inferior blade of DG is seen (Figure 6A: component 2) and an accompanying current source in the granule cell layer (Figure 6A: component 3). For this control mouse, a further small current source is seen in the CA1 pyramidal cell layer, perhaps representing the passive source of a CA1slm sink-source pair (Figure 6A: component 4). In both genotypes a weak sink is visible in CA1 stratum oriens, consistent with synaptic delay from the DG mossy fibres to CA3 and output of CA3 Schaffer collaterals onto basal CA1 dendrites (Figure 6A: component 5). These CSD patterns were typical of first pulse responses for all animals, including those classified as ‘excitable’ (DG PS and/or re-entrance) and in those that did not show any evidence of PS in DG or CA1.

In response to pulse 5, a clear PS could be seen in the DG of the example 3xTgAD animal and this DG population spike also begins to emerge in the control animal (Figure 6, pulse 5 upward arrow). Visible in the control mouse, there was also the appearance of a slightly longer component extending the existing CA1slm sink, perhaps within the region of CA1 where CA3 Schaffer collaterals synapse onto CA1sr (also visible in Figure 6C pulses 10 and 20). It is likely that this response was also present in the selected 3xTgAD mouse; however, the sink was likely obscured by the large DG PS.

In response to pulse 10, long-latency re-entrance (presumably transferring through EC) in the 3xTgAD animal is seen in the form of a sink-source pair in CA1 cell layer at around 15 ms latency (Fig. 6D: component 6). There is still a clear early PS in DG, and there is a long-latency DG source, perhaps resulting from the inferior blade cell layer.

By pulse 20, the example control animal is also showing a clear long-latency CA1 current source; the CA1 response being accompanied by a population spike in the 3xTgAD example (Figure 6D upper black arrow). In the 3xTgAD profile the early DG cell layer response is curtailed, by a presumed inhibitory after-potential (Figure 6D: IAP); however, there is a clear re-entrant population spike in the inferior blade of DG (Figure 6D lower arrow). Figure 6B shows pulse 20 for the 3xTgAD animal in more detail, where the re-entrance of the CA1 and DG response is clearly reflected in the overlaid LFPs. Thus, the 3xTgAD animal appears to demonstrate increased excitability in DG, as indicated by a larger early response to activation of the PP.

To summarize our CSD findings in young animals, we saw a pattern of sources and sinks revealed by CSD analyses that were consistent across controls and 3xTgAD mice with the expected connectivity of the layer II and III EC inputs to the hippocampal formation. In addition, comparative excitability was increased in DG and CA1 of 3xTgADs to both the direct effect of PP activation and indirect, delayed re-entrant input, presumably also arriving via PP afferents.

### 5 Hz train analysis: Old animals of 17–18 months of age

**Figure 7:**
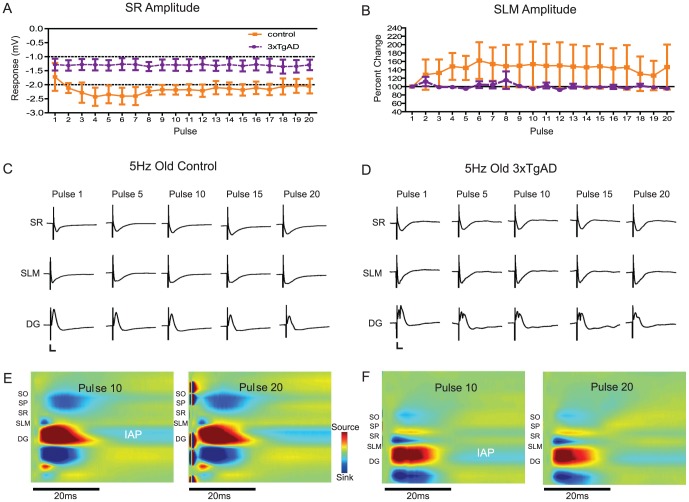
Train stimulation at 5 Hz in old animals. A. There is a significant genotype difference in raw fEPSP amplitude in old animals upon 5(P<0.05). B. CA1 responses did not facilitate following pulse 1 in old 3xTgAD animals (data shown for CA1slm). Control [C] and 3xTgAD [D] representative responses for old animals. Note a lack of increase in response magnitude as the train progresses. 3xTgAD animal in [D] was the only old animal to show DG PS. Calibration 2 mV/10 ms. CSD analysis of pulses 10 and 20 in representative old control [E] and 3xTgAD [F] animals. CSD components are comparable to those seen in young mice at pulse 1 (Fig 6C & D; current sinks in blue, sources in red). A prolonged, facilitating afterpotential is clearly visible from 20 ms in the DG of both control and 3xTgAD animals (marked IAP).

For old animals, there were no significant effects on the amplitude or latency for any response (when analysed as percentage change, i.e., normalized values); however, it is likely that some effects could have been masked due to large variances in the control sample (n = 5 control, n = 3 AD). Nevertheless, analysing ‘raw’ response amplitudes did show genotype differences. In detail;

CA1sr response amplitudes showed a significant effect of genotype for raw amplitude (F(1,114)  = 6.96, P<0.05), with 3xTgAD animals showing little fEPSP facilitation following the first stimulus pulse compared to controls (Figure 7A). There was a trend for controls to show facilitation over the course of the train but this effect did not reach significance. For pulse 20, the 3xTgAD fEPSP (early PP-evoked response) peaked at 9.87±1.64 ms (n = 3) and for control animals at 8.63±1.45 ms (n = 5); however, no long latency, re-entrant responses were seen in either group.

CA1slm responses showed no significant genotype differences for either fEPSP amplitude or latency (data not shown), however, there was again a trend for CA1slm amplitude to remain steady over the train in 3xTgAD mice (Figure 7B). Again, responses in controls appeared to facilitate over the course of the train but this group difference could have been masked by the large variance of the control data set. The CA1slm response to pulse 20 peaked at 6.69±2.75 ms (n = 4) in 3xTgAD and at 7.71±1.26 ms (n = 5) in control mice; however, no long-latency re-entrant responses were seen.

DG responses showed a significant effect of pulse number on response amplitude (F(19, 114)  = 4.20, P<0.0001 raw; F(19, 114)  = 4.67, P<0.0001% change) as pulses depressed over each subsequent trial; however, in contrast to young animals, there was little evidence of DG PS activity (except for one 3xTgAD animal: see Figure 7D). 3xTgAD early responses peaked faster than those for control animals (5.76±1.17 ms (n = 3) versus 7.47±1.95 (n = 5) respectively); however, again there was a large within group variance, perhaps due to an effect of age and sample size. Also, as there were no long-latency DG responses this again supports a lack of re-entrance in all old mice.

Laminar response profiles during pulse trains are shown in Figure 7 panels C (control) and D (3xTgAD). In contrast to the traces seen in young animals (Figure 5 panels C and D), very little facilitation was seen in any cell layer. Thus, in summary, the latencies to the initial response peak/trough in old animals were comparable, if slightly longer (c.1ms) than those in young animals; however, no long-latency response components were seen in CA1 and DG of old mice at delays that would be associated with hippocampal re-entrance in younger animals. The lack of an effect of pulse number in CA1 responses, and of PS activity in animals of both genotypes, likely reflects age-related changes in excitability.

### Current source density analysis: old animals

Mice aged 17–18 months demonstrated a pattern of synaptic current sources and sinks consistent with activation of the EC PP from layers II and III. This profile was similar to that seen in young animals. However, whereas young 3xTgAD and control mice demonstrated increasing excitability over a pulse train, the CSD profiles of old animals remained largely unchanged (Figure 7 panels E and F). Both genotypes showed what appeared to be an inhibitory after-potential in the DG granule cell layer following the source seen in the main DG response (Figure 7 panels E and F). Control mice showed a large sink in CA1 stratum oriens (smaller in 3xTgAD) at a slightly longer latency than the CA1slm response, consistent with a synaptic delay from the CA3 Schaffer collaterals. In the example shown (Figure 7F), the 3xTgAD animal showed a current source in CA1sr/stratum pyramidale.

To summarize the results from aged animals, CSD analyses revealed that the expected synaptic components reflecting PP inputs to hippocampus were present in animals of both genotypes; however, progressive changes in excitability to 5 Hz stimulation were not observed. There was a significant effect of genotype on the absolute response amplitude evoked in CA1sr and a clear trend for a similar lack of facilitation in CA1slm (although, not significant). Thus, we found evidence for decreased short-term potentiation in the form of train responses in old 3xTgAD mice, above the general decline of amplitude seen in control mice in normal aging.

## Discussion

Alzheimer's disease (AD) is characterised phenotypically by a loss of episodic memory and pathologically by the stereotypical accumulation of Aβ and tau protein. We have shown previously that the 3xTg murine model for AD exhibits a selective deficit in hippocampal-dependent, episodic-like memory at 6 months of age with intact but declining performance at 3 months [23], [24]. At six months of age, 3xTgAD mice show pronounced intracellular accumulation of Aβ [20], [21], strongly suggesting a causal role for this AD pathology in our observed episodic deficits. In the present study, we sought to examine whether this episodic-like memory deficit is accompanied by changes in hippocampal synaptic function through *in vivo* electrophysiological recordings in 3xTgAD and control mice. A positive outcome would provide strong evidence for a neural correlate of memory loss in early AD or MCI. To this end, we examined synaptic responsiveness to single pulses of increasing current intensity and short-term synaptic plasticity through PPF and circuit reverberation (re-entrance) in response to train stimulation in 3 regions of the hippocampal circuit: CA1 stratum radiatum, CA1 stratum lacunosum-moleculare and DG.

### Synaptic connectivity and the ‘tri-synaptic’ pathway

We found no significant genotype difference in the amplitude of evoked responses to increasing input currents in 3xTgAD and age-matched control mice at 4–6 or 17–18 months of age. This is in contrast to previous *in vitro* reports in the same model and the APP_SWE_ mouse suggesting a depression in the input/output curve upon the accumulation of intracellular Aβ [21], [31] but agrees with other findings, showing no basal response change [30]. The discrepancies between reports could be due to methodological differences (*in vitro* slice recordings versus *in vivo* recordings in the present study) and/or stimulation protocol. More specifically, in the research cited above, single-pulse responses were examined through direct stimulation of a monosynaptic pathway (Schaffer collateral input to CA1 or PP input to DG [21], [30], [31]). In the current study, responses were generated through stimulation within the molecular layer of the subiculum, creating several discrete responses: (1) an early mono-synaptic (‘direct’) response in DG and CA1slm through activation of *en passage* PP fibres (the EC layer II and III inputs to DG and CA1slm, respectively); (2) di-/tri-synaptic responses in CA1sr and CA1so (presumably via propagation through CA3); and (3) a longer-latency polysynaptic (‘indirect’) response in DG and CA1. The latter represents ‘re-entrance’ of synaptic activity, shown in the rat to depend on activation of subicular/CA1 output that targets deep layers of EC, from where recruitment of deep-to-superficial connectivity within EC then allows ‘re-entrance’ of evoked activity into the DG and hippocampus via the PP [9], [10]. This re-entrant response is most apparent during repetitive stimulation, suggesting that it requires substantial temporal synaptic facilitation within EC. Thus, in our *in vivo* 3xTgAD model, the combination of activating direct and indirect pathways could, perhaps, have offset abnormalities present in the monosynaptic CA3-CA1 pathway of the 3xTgAD as found by Oddo et al. [21]. Due to a lack of any abnormality in the basic synaptic connectivity of the 3xTgAD model shown here *in vivo*, we propose that any differences found in the current study for PPF or trains between the control and 3xTgAD mouse cannot be attributed simply to a lower capacity or conductance of damaged fibre pathways [28] as the current required to elicit a half-maximum fEPSP was not different between genotypes. Thus, in the following sections we discuss evidence for AD-specific changes in short-term synaptic plasticity that are separate from the basal synaptic abnormalities reported previously in the 3xTgAD mouse [21].

### Increased synaptic facilitation in the 3xTgAD model supports the early development of hyper-excitability

To examine short-term synaptic plasticity, mice were subjected to paired-pulse stimulation over a variety of intervals [39]. In DG, the granule cell response to such short interval PPulse stimulation is modulated through a combination of: (a) feed forward, GABAa-dependent inhibition driven via PP input to local interneurons; (b) pre-synaptic metabotropic glutamate receptor activity (mGluR;[40], [41]); and (c) feedback inhibition elicited from recurrent interneuron collaterals in the vicinity of the activated granule cell [41], [42]. The net effect of these mechanisms is that DG responses normally show paired-pulse depression to PP activation. In the present study, control mice, as expected [42], exhibited paired-pulse depression to short PPIs (25–50 ms). This depression is presumably produced through polysynaptic GABAergic feedback, driven by the first pulse, causing hyperpolarisation of GCs (mediated by GABA_A_ and GABA_B_ receptors[43], [44]). This inhibitory modulation not only results in depression of the field response to PPulse stimulation, but also depresses the GC PS to the second pulse of the pair; this effect wanes as the hyperpolarising current decays during longer PPulse stimulus intervals (from 100 ms onwards; [45]). In agreement with the above mechanism, we found control mice were most likely to display a PS to the second pulse of a pair at PPIs of 100 ms or more, that is, at longer intervals where presumably inhibition has subsided. In contrast, we found the PPulse response profile of 3xTgAD mice in DG was quite different from that of controls. Young and old 3xTgAD mice showed pronounced synaptic facilitation at short PPIs (25 and 50 ms), often accompanied by a PS to the second pulse. In particular, 3xTgAD mice were more likely to display a DG PS to the second pulse at short inter-stimulus intervals with an apparent increasing suppression of PS at longer paired-pulse intervals (that in controls were more likely to elicit PS). We suggest that these results demonstrate a long-term shift to hyper-excitability in the layer II input to DG in these AD mice for high-frequency inputs. In contrast, we saw a shift to increased depression for low-frequency inputs at longer PPI and in the train stimuli. Overall, these data suggest that DG in 3xTgADs may also show a more pronounced ‘late’ inhibitory component compared to controls, perhaps representing a homoeostatic compensatory mechanism to ameliorate the impact of reduced ‘early’ inhibition. Such an increase in late inhibition may also underlie the poor facilitation shown by 3xTgADs to low-frequency train stimulation (see below).

When examining PPulse responses in CA1 sr and slm, we found no overall genotype differences in PPF, and CA1 responses facilitated relative to the first pulse at short intervals in accordance with previous reports in young AD mice [30], [46]. However, we found 3xTgAD animals demonstrated significantly more facilitation in CA1sr relative to controls at a PPI of 50 ms, in accordance with the findings of Gengler et al at the same age [46]. In old animals, the CA1sr response of both genotypes was shown to facilitate at short intervals but, again, there was a significantly increased CA1sr response in old 3xTgAD animals at PPI 50 ms. Thus, it appears from examining the responses of DG (fEPSP and PS data), that there is a subtle reduction in the short-term, recurrent inhibitory feedback system in the 3xTgAD mouse at short PPIs. It is also possible that an excess of excitability in DG and subsequent polysynaptic propagation of ‘overly excitatory’ responses through CA3 to CA1sr could account for the facilitation that we witnessed in CA1sr. Such a mechanism could contribute to aberrant excitation and seizure-like activity, phenomena witnessed *in vivo* in other AD mouse models [33]–[35].

### Mechanisms for increased short-term excitability

Due to the unexpected differences between control and 3xTgAD mice (i.e., the facilitation seen in 3xTgAD PPulse responses), it is worthwhile discussing mechanisms though which the AD phenotype could exert a hyper-excitable effect. There is little evidence for intra or extracellular Aβ pathology in the DG of the 3xTgAD model and no tau pathology localised to the DG in older animals [20], [21]. This would suggest that any change in inhibition is separate from these signature AD pathologies. Indeed, in human AD, the DG is one of the hippocampal regions least affected by pathology [1]. Previous research in other AD mouse models (APP/PS1, APOE ε4 and hAPP J20) has demonstrated a loss of interneurons within CA1 and DG. Specifically, those expressing calcium binding proteins parvalbumin, calbindin and calretinin, resulting in alterations in GABAergic interneuron populations and paralleling interneuron loss in human AD patients [47]–[49]. Recently, degeneration of GABAergic interneurons in DG expressing Neuropeptide Y was seen in a novel triple-transgenic AD model (TauPS2APP mouse; [50]). Interestingly, enhanced LTP to PP stimulation was seen in this triple mutation mouse in DG (*in vitro*), therefore, it is possible that loss of GABAergic tone in this and other models could contribute to hyper-excitability originating in DG [33]–[35], [50]. Although this could account for the excessive facilitation seen in DG and CA1sr, we know from immunohistological studies that the 3xTgAD mouse model does not experience overt cell loss [20], [21]. Thus, there may be similar mechanisms present in the 3xTgAD mouse detectable through the application of antibodies sensitive to GABA interneurons expressing calcium-binding proteins. Alternative/additional factors include the abnormal calcium homeostasis linked to PS1mutations [26], [51], [52], a reduction in nicotinic α7 acetylcholine receptors (α7nAchRs [53]) and abnormalities in the mGluR I-III families [54], [55].

### 3xTgAD field responses to low-frequency pulse trains are depressed; however laminar synaptic connectivity profiles appear normal and response re-entrance into the hippocampal circuit is enhanced

Alongside responses to single and PPulse stimulation, we examined the effects of low-frequency trains on presumed monosynaptic and polysynaptic/re-entrant CA1 and DG responses [7], [11]–[13]. Young mice displayed synaptic excitability (increased DG population cell spiking) and reverberation within the hippocampal formation, in a subset of both 3xTgAD and control animals. This confirms for the first time the presence of hippocampal output and re-entrant pathways in the mouse, as seen previously in rat ([9], [10]. As an effect of genotype, small changes in fEPSPs could be seen in CA1sr (and to a lesser extent in CA1slm) in young 3xTgAD mice. Over a train of 20 pulses, the fEPSP amplitude of 3xTgAD mice tended not to facilitate as much as seen in controls (although there were no significant genotype differences), further, they displayed relatively constant response latencies over the train where response latencies in control mice generally slowed. In the 3xTgAD mouse, this effect was independent of response amplitude, thus, represented a faster rise time to fEPSP peak.

CSD profiles in both 3xTgAD and control mice to train stimulation showed a pattern of current sinks and sources consistent with excitatory synapses onto DG molecular layer (GC dendrites) and apical dendrites in CA1 [9], [56]. If the connectivity between subiculum, EC and hippocampus was intact in the early stages of AD we expected to see: 1) a current sink in DG, reflecting layer II EC PP inputs to the dendrites of granule cells within the molecular layer and a positive-going fEPSP (presumably a reversal of the population cell fEPSP); 2) a concomitant current sink (reflected by a fast, negative-going local field potential; LFP) within CA1slm, marking the excitatory input from layer III PP (stimulated directly by the traversing of fibres through subiculum); 3) a delayed CA1sr negative going LFP, of a latency reflecting synaptic delay through the tri-synaptic connectivity to EC, DG, CA3 and ending with the Schaffer collateral input to CA1sr [9], [10].

The presence of a clear CA1slm sink was variable in both genotypes, likely due to the relative success of electrode placement within the narrow CA1slm layer, rather than an abnormality in the PP input. In all animals, a large DG current source could be seen upon population spiking, thus, synaptic transmission through PP layer II appeared intact in the 3xTgAD mouse. CSD sinks also indicated that the (presumed) Schaffer collateral input from CA3 terminated in both stratum oriens and stratum radiatum of CA1, suggesting that the connectivity of hippocampal fibre pathways are functionally intact in the 3xTgAD mouse (but see [28]). Note that we cannot discount a contribution from CA2 input to CA1 so/sr [57]. However, the CA1sr latency would appear rather too long for a di-synaptic response (EC layer III input to CA2 which then projects to CA1 so/sr). Thus, the CA1sr response is much more likely to reflect feed-forward excitation from DG via CA3 [9], [10].

Animals that demonstrated response re-entrance also showed progressive DG (and CA1 in 3xTgAD) population cell spiking and, within a train of 20 pulses, there was evidence for hippocampal excitability within each layer of the CA1-DG axis. For 3xTgAD in particular, the re-entrant input to CA1 elicited PS, which could be seen within as few as ten pulses of a train. However, 3xTgAD excitatory responses were also frequently coupled with what were presumably prolonged inhibitory after potentials (also seen in non-excitable 3xTgAD mice), perhaps indicating abnormalities in the balance of inhibitory feedback within hippocampus [33]. The presence of putative inhibitory currents during a 5 Hz train is supported by the lack of a DG second PS during seen PPulse stimulation, where a 100 ms or more ISI suppressed DG PS activity in 3xTgADs.

The highly specific topographic connectivity of the hippocampal-entorhinal system [8]–[10], [58]–[60] may also have meant that subtle differences in recording and stimulating placements produced a range of response profiles within each genotype; however, placement of electrodes was consistent across all animals at the targeted coordinates. Thus, the present response profiles strongly suggest that there are subtle differences in the laminar profile of hippocampal responses in 3xTgAD and control mice, in response to low-frequency stimuli.

By 17–18 months of age, we found significant differences in the size of CA1sr fEPSP amplitude generated in the 3xTgAD mouse; further, their CA1 responses did not facilitate relative to the first pulse of the train. This effect appeared to be independent of the normal ageing process, as control mice retained some degree of facilitation; therefore, we conclude that this change in the 3xTgAD mouse is due to AD-like pathological progression. The latter likely includes the appearance of extracellular plaques and hyperphosphorylated tau [20], [21] in addition to a range of other pathological markers, including increase in de-myelination [28], inflammatory processes [20] and/or loss of synaptic density [32]. There was, however, a general decrease in excitability with age in both genotypes, which could be due to an age-related increase in inhibitory after-potentials, such as those after-hyperpolarisation currents seen when recording intracellularly from hippocampal slices derived from old animals [61]. The latter conclusion is supported by the CSD profiles of aged animals which appear to show: (a) a sustained sink current in DG; (b) the lack of hippocampal re-entrance; and (c) a decrease in PS probability during both PPulse and train stimulation. Due to the small numbers of animals in the old group, conclusions must be drawn with caution; nevertheless, it appears in the aged 3xTgAD mouse there is augmentation of the usual decline in synaptic facilitation associated with ageing. Of note, there is also an age-related decrease in DG paired-pulse depression in control mice, a change that may underlie the known ageing-related deficit in pattern separation (see [62] for review) and, perhaps in turn, the age-related deficit in episodic-like memory in older mice [23].

## Conclusions

What do the present data contribute to our understanding of early hippocampal dysfunction in AD? We found previously that the 3xTgAD model exhibits an early deficit in episodic-like memory that develops fully by 6 months of age [23], [24]. We show here that this behavioural deficit coincides with subtle abnormalities in neuronal excitability and paucity of fEPSP facilitation seen in the DG and CA1 regions, suggesting an imbalance of inhibitory feedback in the model. However, our single-pulse responses show no obvious evidence of degradation in hippocampal fibre pathways [28]. Therefore, we suggest that cellular/synaptic abnormalities (be it intracellular Aβ accumulation, abnormal calcium homeostasis or dysfunction of recurrent inhibition) are mediating the effects witnessed here, at least in young mice. These findings do not necessarily contradict deficits seen in LTP (which are likely interlinked with intracellular mechanisms) seen in this and other AD mouse models [21], [25], [32], [46]. However, a major conclusion from the present experiments is that hippocampal synaptic integrity in the 3xTgAD model of familial human AD is largely intact (as measured through single and paired-pulse low frequency stimulation *in vivo*) but that responses to brief high-frequency activation display hyper-excitability. If the 3xTgAD model does indeed mirror the pathological progression of familial human AD (bearing in mind that no familial patient would experience multiple mutations in APP or PS1 together as in this AD model), these results provide some hope that in the early stages, the hippocampus is functionally complete. Thus, treatments that can alleviate intracellular abnormalities that underlie AD-related synaptic changes (such as chelation of excess calcium; [26] or clearance of intracellular Aβ [22]) may improve cognitive function and quality of life in individuals at the onset of AD by correcting the hippocampal hyper-excitability shown here by us and others in AD models.
